# Identifying user profiles of healthcare, social and employment services in a working-age population: A cluster analysis with linked individual-level register data from Finland

**DOI:** 10.1371/journal.pone.0293622

**Published:** 2023-11-01

**Authors:** Jenni Blomgren, Sauli Jäppinen, Riku Perhoniemi

**Affiliations:** 1 Research Unit, The Social Insurance Institution of Finland, Helsinki, Finland; 2 Analytics Unit, The Social Insurance Institution of Finland, Helsinki, Finland; Tampere University, FINLAND

## Abstract

A thorough understanding of the use of services in the population is important in order to comprehend the varying service needs of different groups. This explorative study aimed to find distinct user profiles in a working-age population based on individuals’ annual use of healthcare, social and employment services and to explore socio-demographic and morbidity-related predictors of the user groups. Administrative register data on the use of various services and individual-level covariates from year 2018 were linked for all residents aged 18–64 of the municipality of Oulu, Finland (N = 119,740). K-means cluster analysis was used to group the study subjects into clusters, based on their frequency of using 22 distinct healthcare, social and employment services during 2018. Multinomial logistic regression models were utilized to assess the associations of cluster assignment with socio-demographic and health-related covariates (sex, age, marital status, education, occupational class, income, days in employment, chronic disease and receipt of different social benefits). Five distinct clusters were identified in terms of service use, labelled low to moderate users of healthcare (82.0%), regular employment services users with moderate use of healthcare (9.6%), supported employment services users with moderate use of healthcare with an emphasis on preventive care (2.9%), frequent users of healthcare, social and employment services (2.9%), and rehabilitation, disability services and specialized healthcare users (2.6%). Each cluster not only showed different patterns of service use but were also differently associated with demographic, socio-economic and morbidity-related covariates, creating distinct service user types. Knowledge on the different user profiles and their determinants may help predict future need and use of services in a population, plan timely, coordinated and integrated services, and design early interventions and prevention measures. This is important in order to save costs and improve the effectiveness of services for groups with different care needs.

## Introduction

In-depth understanding of individuals’ use of healthcare and social services is needed in order to design the most suitable service systems for different population groups. Information on different user profiles and care needs is crucial for managing and coordinating integrated services, developing interventions and prevention strategies, containing costs and improving patient outcomes [1–3]. In the research literature, the main interest has often been in population groups that use a lot of healthcare services. Several studies from Western countries have sought to identify so-called frequent attenders or high-cost users of healthcare and aimed to find predictors of high utilization. Studies have commonly found that a small part of the population uses most of the services and incurs most of the costs of care, and that this phenomenon is associated with factors such as higher age, being female, chronic conditions, complex health problems, mental health issues, low socio-economic status and other adverse social factors [2–15].

The main potential in coordinating services and containing costs may indeed lie in the high-cost frequent attenders. However, it is also important to identify groups with limited but specific care needs and those that rarely or never use services, as carefully planned services may prevent them from not burdening the service system and becoming high-cost frequent attenders later on [1,2]. Furthermore, knowledge on how various individual-level covariates are associated with different user groups is crucial as it helps in identifying population groups with different care needs, and thus aids in planning suitable services and in coordinating care packages.

Many previous studies have used cluster analysis techniques to identify homogeneous latent groups of users of health services, and to assess the determinants of different user groups [1,16–20]. Studies utilizing clustering have often found one or two large clusters of persons with low service use, one or several high and/or multiple service use clusters, and a couple of clusters with lower levels of service use but concentrated on some services only [1,16,17,19]. A common limitation in previous studies is that they have tended to include only one service type or a limited set of services, and usually only healthcare services. However, an individual’s entire service spectrum, including both healthcare and social services as well as employment services, needs to be taken into account as comprehensively as possible to be able to truly design effective integrated services for different population groups.

The aim of this study was twofold: 1) to find whether meaningful distinct groups can be identified within the working-age population in Finland in terms of the individuals’ total use of healthcare services as well as social and employment services during one year, utilizing cluster analysis, and 2) to find how various socio-demographic and morbidity-related covariates are associated with assignment to each cluster. The study concentrated on working-age persons because of inherent differences in service schemes available for different age groups.

## Materials and methods

### Register data linkages and study population

The study utilizes an individual-level data set collected for research purposes from administrative registers and linked for the population of the municipality of Oulu, Finland, for the research project ‘Social and health care services and social security benefits in Oulu in 2013–2018’ [21]. Oulu is the fifth largest municipality of Finland, with approximately 204,000 inhabitants at the end of 2018 [22]. The study population for this study was identified using register data from the Social Insurance Institution of Finland (Kela). It consisted of working-age persons (age 18–64) who were residents of Oulu both at the beginning and at the end of year 2018 (N = 119,740). Individual-level register data were linked from Kela, the municipality of Oulu, the National Institute for Health and Welfare, four occupational healthcare (OHC) providers, the Ministry of Employment and the Economy, the Finnish Centre for Pensions, Statistics Finland and Finnish Tax Administration (for more information on the data set, see [21]). All data sets retrieved from separate register holders were linked at the individual level by the means of a pseudonymized personal identifier. The researchers did not have access to actual identifiers enabling disclosure of identity. According to regulations governing scientific research in Finland, the study did not require informed consent or institutional ethics review board approval since only pseudonymized administrative register data were used and the study subjects were not contacted to collect the data [23].

### Service variables used in clustering

This study utilized register data on use of healthcare, social and employment services incorporated in the Finnish social security system (for descriptions of the Finnish social security and service system, see, for example, [24–26]). Healthcare and social services form the core of the service structure. The care system in Finland is based on universal coverage of public healthcare and social care for every citizen. In 2018, i.e. the focal year of this study, municipalities were responsible for organizing services for their inhabitants, including primary and secondary healthcare as well as social welfare services. In addition to public services, employed persons are entitled to employer-provided OHC, often including both statutory preventive services as well as curative primary care, and that are free of charge for the user at the point of delivery. Also private sector healthcare, partly compensated by National Health Insurance (NHI), is available for those with the ability to pay high co-payments. In addition, employment services are offered by the employment administration to job seekers. Along with regular employment services for those with lesser needs, supported employment services (often resembling social services of the municipalities) are targeted to those who have special needs and difficulties in finding employment or who have work ability problems.

**Fig 1:**
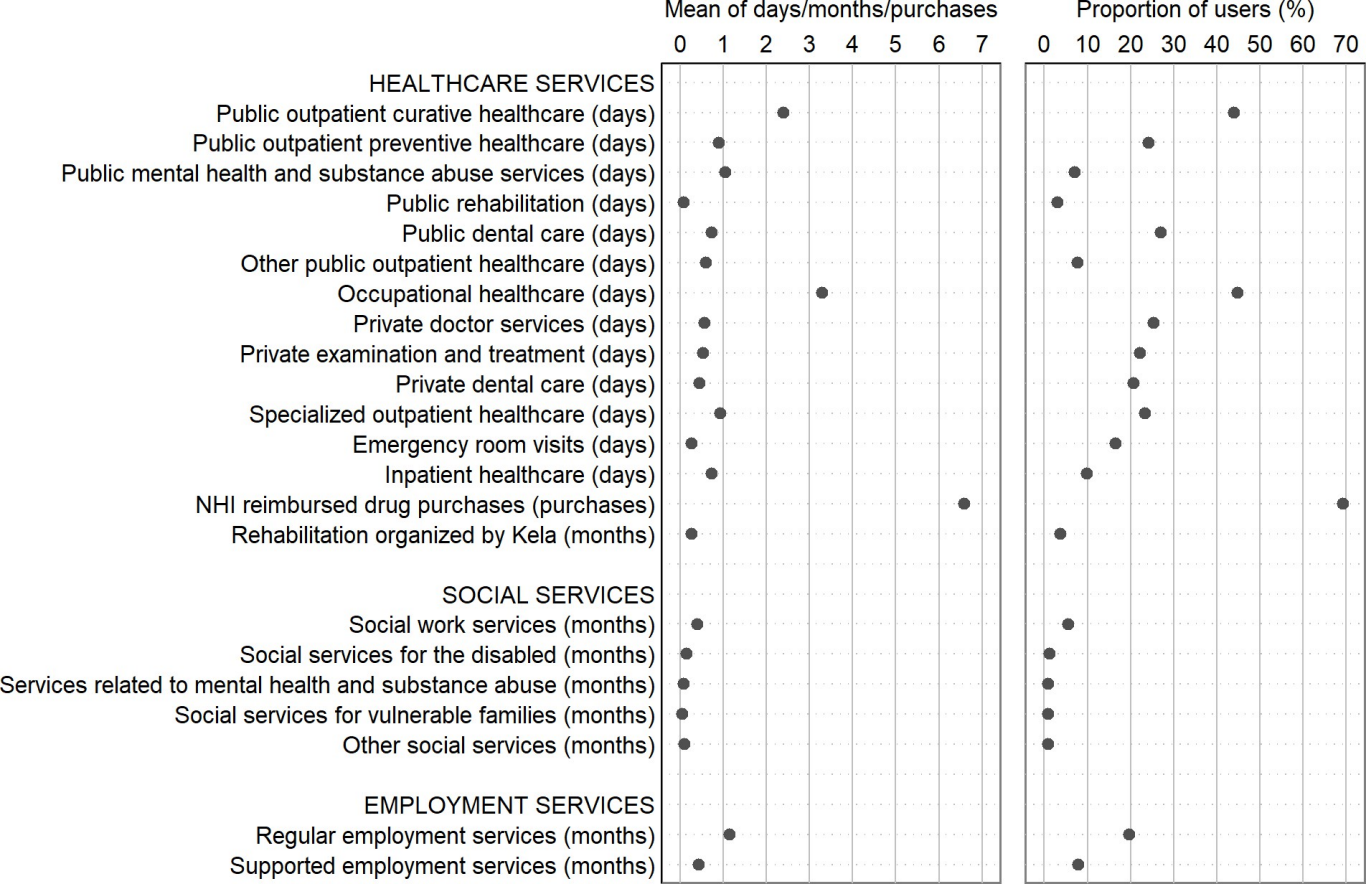
Distribution of usage of healthcare, social, and employment services in the study population, Oulu, Finland, 2018. NHI = National Health Insurance.

The specific service types identified in this study (N = 22) are shown in Fig 1, with their means and proportions of users in the study population during 2018. Healthcare services included in the study comprise services organized at public, occupational and private healthcare schemes, and include both outpatient and inpatient healthcare. Data on use of public outpatient healthcare services were collected from the municipality of Oulu and register of outpatient healthcare maintained by the National Institute for Health and Welfare, data on private healthcare from the registers of Kela, and data on occupational healthcare from four largest providers of OHC in Oulu, which were, at the time of data collection, Attendo, Mehiläinen, Terveystalo and Työterveys Virta. These four OHC providers taken together have been estimated to cover over 90% of all OHC in Oulu [27]. Data on specialized outpatient healthcare, emergency room visits and inpatient care episodes were retrieved from the registers of the National Institute for Health and Welfare. Furthermore NHI-reimbursed drug purchases as well as rehabilitation periods organized by Kela were included as additional variables depicting healthcare use and were retrieved from Kela’s registers.

Data on social services (five different service types) were retrieved from the registers of the municipality of Oulu, and data on employment services (two service types, distinguishing being a customer of regular vs. supported employment services) from the register of the Ministry of Employment and the Economy (Fig 1).

Use of healthcare services was measured as distinct days of using each service during year 2018. However, NHI reimbursed drug purchases were measured as separate purchases of each medicine, and participation in rehabilitation organized by Kela was measured as distinct months, since measuring distinct rehabilitation days was not possible. Also use of social services was measured in distinct months, since in the original data sets, access to these services was defined on a monthly basis through being a customer of each service during a given month. Likewise, being a customer of employment services was measured in distinct months.

### Socio-demographic covariates, chronic disease and benefit receipt

All predictors were measured at the beginning or during year 2018. The distributions of covariates are shown in Table 1. Demographic covariates were retrieved from the register of Kela and included sex (male; female), age (18–24; 25–34; 35–44; 45–54; 55–64) and marital status (married; never married or marital status missing; divorced or widowed).

**Table 1:** Distributions of the covariates in the study population.

Variable ^1^	Distribution (%)
**Sex**	
Male	51.4
Female	48.6
**Age group**	
18–24	16.8
25–34	23.7
35–44	22.0
45–54	19.0
55–64	18.4
**Marital status**	
Married	40.6
Never married or missing	47.8
Divorced or widowed	11.5
**Level of education**	
Tertiary	40.3
Secondary	46.9
Basic	12.8
**Occupational class**	
Upper non-manual employee	19.6
Lower non-manual employee	25.3
Manual worker	17.2
Entrepreneur	4.7
Unemployed	10.8
Student	10.9
Other	11.5
**Income (during 2018)**	
Quintile 5 (Over € 47,164)	20.0
Quintile 4 (€ 33,550–47,164)	20.0
Quintile 3 (€ 22,839–33,549)	20.0
Quintile 2 (€ 10,577–22,838)	20.0
Quintile 1 (€ 0–10,576)	20.0
**Time in employment (during 2018)**	
Full year	56.6
Less than a year	23.2
None	20.2
**Number of chronic diseases (during 2018)**	
0	78.2
1	13.4
2 or more	8.4
**Receipt of benefits (during 2018)** ^**2**^	
Unemployment benefit (yes)	20.5
Disability pension or old-age pension (yes)	11.2
Sickness allowance (yes)	9.5
Disability allowance (yes)	3.1
Housing support (yes)	25.8
Basic income support (yes)	8.4
**Total**	100
**N**	119,740

^1^ Variables were measured at the beginning of year 2018 if not stated otherwise.

^2^ Proportions are given separately for receipt of each benefit (non-recipients as complements).

Socio-economic covariates included the level of education (tertiary; secondary; basic) retrieved from the register of Statistics Finland, occupational class (upper non-manual employee; lower non-manual employee; manual worker; entrepreneur; unemployed; student; other) retrieved from the register of Statistics Finland, individual yearly gross income (in quintiles, with cut points of 10,576€; 22,838€; 33,549€ and 47,164€) retrieved from the register of Finnish Tax Administration, and time spent in employment during 2018 (full year; less than a year; no time in employment) retrieved from the register of Finnish Centre for Pensions.

The prevalence of chronic diseases was measured through special entitlements to reimbursement of medicines due to chronic disease (0 diseases; 1 disease; 2 or more diseases), retrieved from the register of Kela. These entitlements may be granted if supported by a physician’s certificate and they guarantee that the patient can obtain free or cost-subsidized drugs needed for treatment for certain long-term and severe diseases. These entitlements may be used for research purposes as a proxy measure for chronic disease [28].

Finally, data on receipt of various social security benefits, i.e. unemployment benefit, disability pension or old-age pension, sickness allowance (for those who are temporarily not able to work due to illness or injury), disability allowance (to cover the additional costs caused by disability), housing support (to cover housing costs for those with low income) and basic income support (for those with little or no other income) were retrieved from registers of Kela and Finnish Centre for Pensions. Information about receiving benefits deepens the understanding of the social status and income sources of the study population. For a detailed description of different benefits, see [24].

### Statistical methods

We utilized cluster analysis to group the study population into latent groups by the individuals’ use of healthcare, social and employment services. Cluster analysis is a statistical technique for revealing latent structures in large data sets by grouping similar individuals into homogenous entities [e.g. 29,30]. The individual-level frequencies of using each of the 22 service types identified in this study during year 2018 were utilized in forming the clusters of service use. As the type and unit of measurement varied between the different service variables (days, purchases or months, see Fig 1), the variables were first transformed to a comparable minimum–maximum scale, ranging between the interval 0.0 to 1.0 [30].

K-means cluster analysis, a commonly used method in this type of studies [1,17,18,20] was chosen as the clustering method. Some advantages of the K-means cluster analyses are that it is fast and efficient in processing large datasets–however, it requires the user to specify the number of clusters [30]. K-means cluster analysis was tested for solutions including 2–6 clusters. Then, the optimal number of clusters was chosen based on cluster metrics (average silhouette width and Calinski–Harabasz index [31]), reasonable cluster sizes and meaningful cluster identities. Comparing the indexes suggested the best average silhouette width (0.7) for the five-cluster solution, supported by the Calinski–Harabasz index that suggested a better fit for the five-cluster than the four- or six-cluster solutions (see S1 Table). Supported by the cluster metrics, the five-cluster solution was finally chosen, as it also provided meaningful differentiation between the clusters and produced interpretable results and distinct cluster identities.

Means for the use and proportions of users of each service type during 2018 were calculated for each cluster. Subsequently, associations of the covariates with cluster assignment were assessed with cross-tabulations and Chi squared tests. Finally, multinomial logistic regression modeling was used to examine the adjusted associations of covariates with cluster membership. In these models, all covariates were adjusted for simultaneously. To ease comparison of the associations, the results were transferred to average marginal effects (AME) with their 95% confidence intervals (CI). The AMEs are on the scale of percentage points and indicate the absolute differences in the predicted probability of cluster membership by covariates, i.e. how many percentage points higher or lower the predicted probability of a certain cluster membership was in a given class of covariate compared to the reference group of that covariate. K-means cluster analysis was conducted with R version 4.2.3 [32] and multinomial regression analyses with Stata version 14 [33].

## Results

### The identified clusters of health and social services users

**Fig 2:**
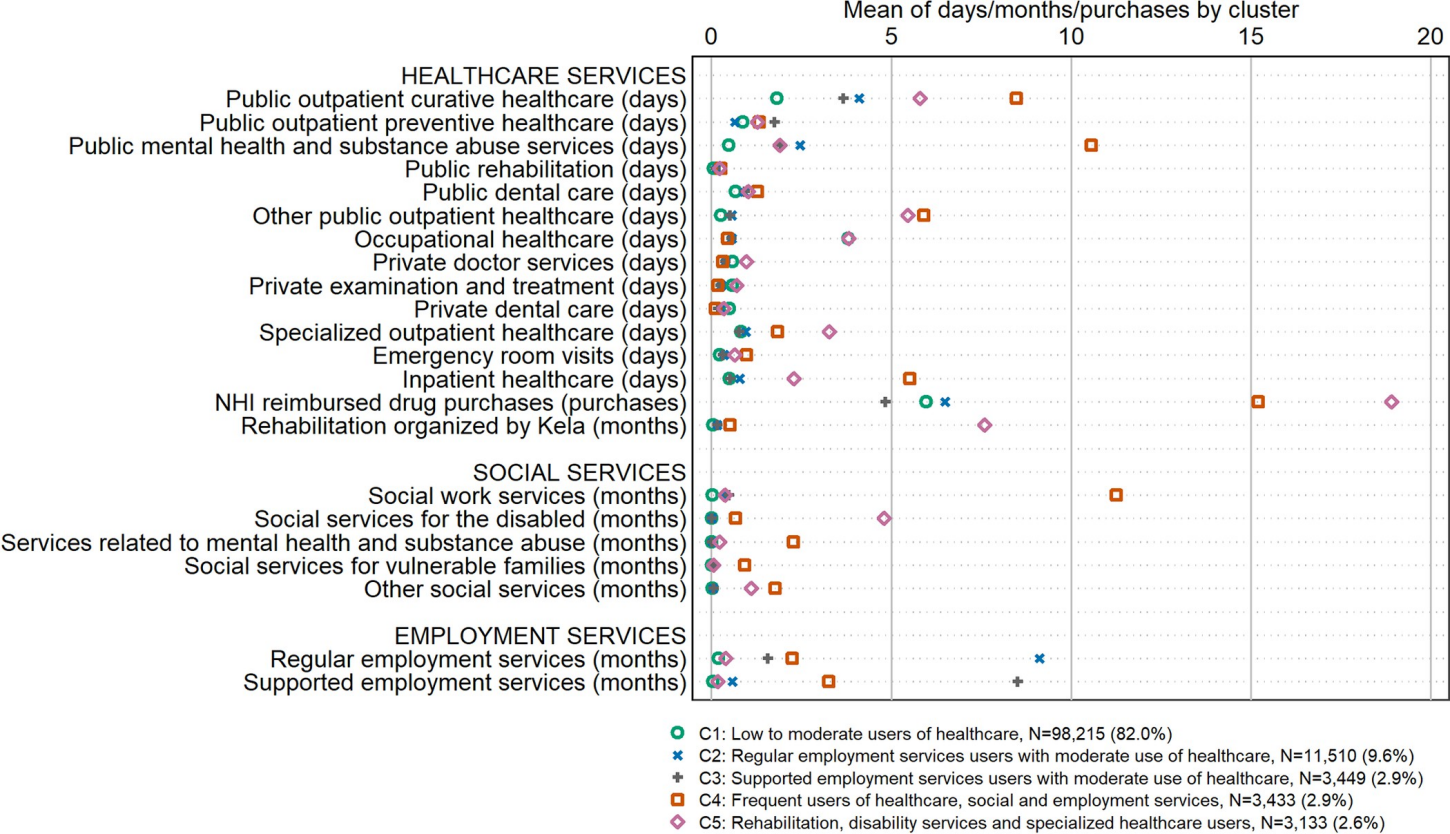
Average frequency of service use in the study population in year 2018 by cluster. NHI = National Health Insurance.

**Fig 3:**
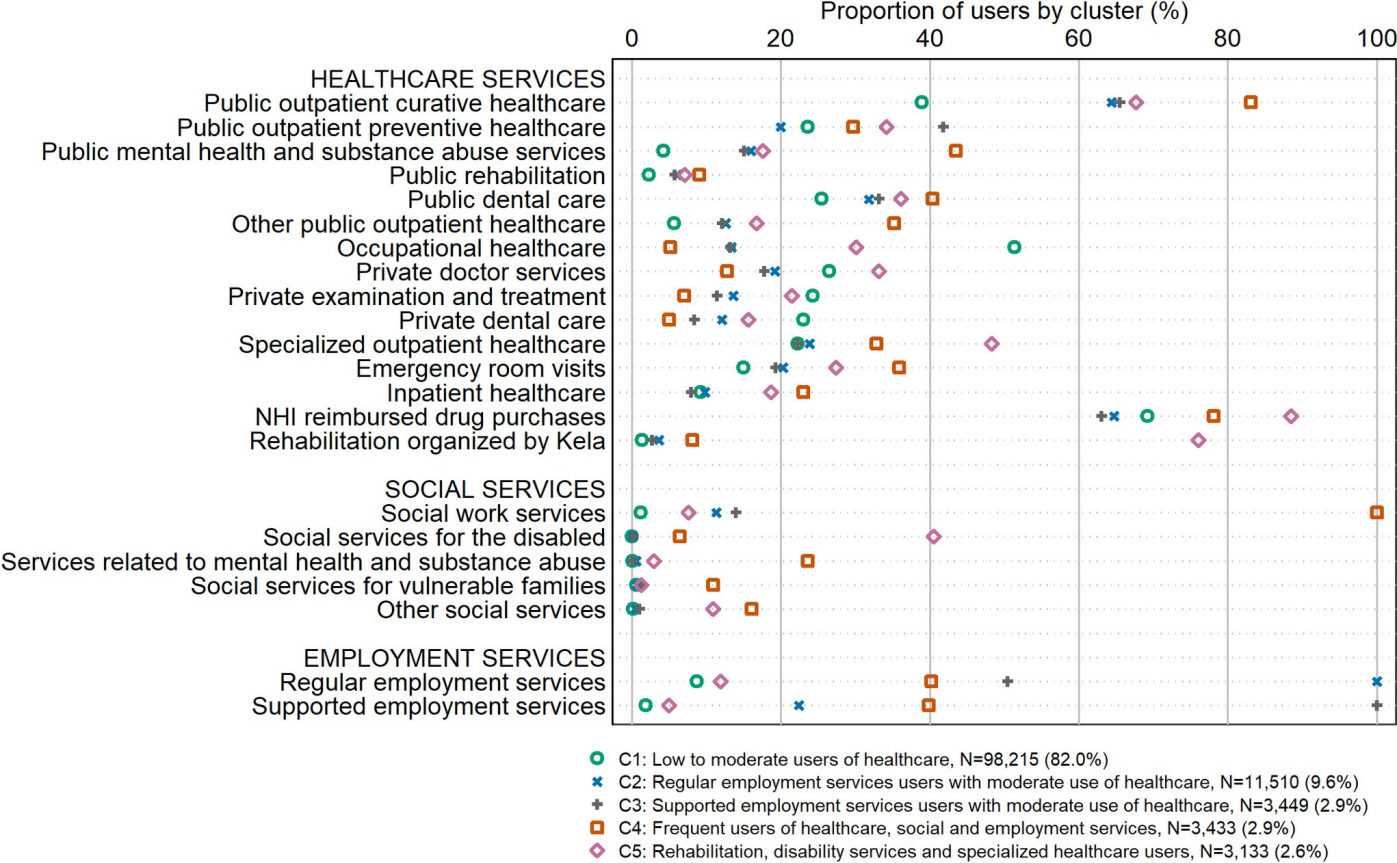
Proportions of users of services in year 2018 by cluster (%). NHI = National Health Insurance.

The five-cluster solution distinguished between clear cluster identities and produced meaningfully interpretable clusters that varied in the persons’ use of the 22 included healthcare, social and employment services (Figs 2 and 3). The clusters were named (1) *low to moderate users of healthcare* (82.0% of the study population), (2) *regular employment services users with moderate use of healthcare* (9.6%), (3) *supported employment services users with moderate use of healthcare with an emphasis on preventive care* (2.9%), (4) *frequent users of healthcare*, *social and employment services* (2.9%), and (5) *rehabilitation*, *disability services and specialized healthcare users* (2.6%). Fig 2 shows the mean yearly number of distinct user days, NHI reimbursed drug purchases or months being a customer of the given service by cluster. Fig 3 shows the proportions of persons using each of the different 22 service types by cluster.

Those who were assigned to cluster 1 (N = 98,215; 82.0% of the study population) used public outpatient curative healthcare clearly less frequently compared to other clusters. In this cluster, also use of heavier healthcare services, such as mental healthcare and substance abuse services, specialized healthcare, emergency room services, inpatient healthcare and rehabilitation services, was at a lower-than-average level. Furthermore, only a marginal proportion used any of the social services, and also employment services were relatively rarely used. However, over one half of persons assigned to this cluster used occupational healthcare services (a clearly larger share than in any other cluster), with on average 3.8 visiting days during the study year. Also use of private doctor and private examination and treatment services was at a slightly higher-than-average level. More detailed analyses (results not shown) showed that all those not using any of the 22 services during the year (N = 6,917; 5.8% of study population) were assigned to this cluster.

Persons assigned to clusters 2 (N = 11,510; 9.6%) and 3 (N = 3,449; 2.9%) were rather similar to each other in terms of their use of healthcare services but they clearly differed from each other in terms of the type of employment services. All persons assigned to cluster 2, i.e. the larger cluster of these two clusters, had been customers of regular employment services during the study year (on average, for 9.1 months during the year)–however, the result does not imply that all those using regular employment services were assigned to this cluster. In contrast, all those in cluster 3 had been customers of supported employment services (on average, for 8.5 months during the year). Use of social services was at a higher-than-average level in these two clusters: 11% persons in cluster 2 and 14% of persons in cluster 3 had been customers of social work services. Persons in these two clusters used public outpatient curative healthcare, public mental healthcare and substance abuse services as well as many other public outpatient services more frequently than those in cluster 1 but less frequently than those in the remaining two clusters. However, persons in clusters 2 and 3 only rarely used OHC services and also the use of private healthcare services was at a lower-than-average level. Some nuances in healthcare use can be seen when comparing clusters 2 and 3: for example, those in cluster 3 used less inpatient healthcare and had on average less drug purchases compared to those in the other clusters. On the other hand, persons in this cluster used more public outpatient preventive healthcare services than any of the other clusters–while use of these services was at its lowest level among those in cluster 2.

Cluster 4 (N = 3,433; 2.9% of the population) was characterized by having the clearly highest overall intensity of using many of the measured healthcare (especially public healthcare), social and employment services. All persons assigned to this cluster were customers of social work services, and 40% were customers of supported employment services. Use of mental health and substance abuse services was frequent, both within healthcare services and within social services. Furthermore, those in this cluster frequently used public outpatient curative healthcare, emergency room services and inpatient healthcare, and the number of compensated drug purchases (15.2) was more than double compared to the average.

Finally, cluster 5 (N = 3,133; 2.6% of the population) consisted of persons using many of the more intensive healthcare services, such as rehabilitation services, specialized outpatient healthcare services and social services for the disabled, on average more frequently than those assigned to the other clusters. However, they were not strikingly often customers of employment services. Also use of occupational healthcare and private healthcare was rather common in this cluster, and the average number of yearly NHI reimbursed drug purchases was highest in this cluster.

### Associations of covariates with cluster membership

Distributions of covariates according to clusters are shown in S2 Table. All associations, tested with Chi squared test, were highly statistically significant at the p<0.001 level. Clusters 1, 2 and 4 consisted more of males than females, whereas cluster 3 and especially cluster 5 consisted predominantly of women. Those assigned to clusters 1 and 2 were on average older than those in other clusters, while especially cluster 3 consisted predominantly of persons aged less than 35 years. Compared to other clusters, cluster 1 included the highest proportion of persons who were married, had a tertiary education, had an occupational status of an upper or lower non-manual employee, manual worker or entrepreneur, had a high income and were in employment for the full year. The average socio-demographic background among those assigned to clusters 2–5 was clearly less advantaged than among those in cluster 1. Cluster 2 consisted mainly of persons having an occupational class of unemployed, while almost a half of those in cluster 3 were classified as students at the beginning of the measurement year even though also the share or unemployed persons was rather high in cluster 3. Especially cluster 4 comprised a high proportion of persons who had never been married, who had only basic level education, whose occupational class was associated with being outside the labour force, who had low income and who had no employment days at all during the year. On the other hand, the proportion of persons with chronic diseases was at its highest level in cluster 5, even though this proportion was higher than average also in cluster 4.

The clusters were clearly different from each other also regarding receipt of different benefits–which is, naturally, associated with the specific service types that characterized each of the clusters. Notably, receiving housing support or basic income support, which are benefits that compensate for otherwise low income, was at a low level in cluster 1. In contrast, those assigned to other clusters and especially those assigned to cluster 4 commonly received these two benefits, which highlights their overall disadvantaged position. The clear majority of those assigned to clusters 2 and 3 and almost half of those in cluster 4 had received unemployment benefit during the year. On the other hand, receipt of pensions (mainly disability pension in this age group), sickness allowance and disability allowance was most common in clusters 4 and 5.

Table 2 shows the fully adjusted average marginal effects (AME) of being assigned to each cluster by covariates, retrieved from multinomial logistic regression models. Adjusted associations between cluster assignment and covariates were partly different from the unadjusted ones. Generally, when looking at the effect sizes in the adjusted results, sex, marital status, level of education and chronic diseases were not as strongly associated with cluster membership as the other variables in the model. After mutual adjustments, the likelihood to be assigned to cluster 1 was lower in other age groups compared to the youngest group, and also decreased especially by being unemployed by occupational class, having lower income, not being in employment the full year and having chronic disease. Being a recipient of any of the included benefits, most notably unemployment or disability benefits, decreased the likelihood to be assigned to this cluster.

**Table 2:** Predictors of the clusters defined by use of healthcare, social and employment services.

	Clusters of services users									
	1. Low to moderate users of healthcare		2. Regular employment services users		3. Supported employment services users		4. Frequent users of different services		5. Rehabilitation and disability services users	
**Variable**	AME	[95% CI]	AME	[95% CI]	AME	[95% CI]	AME	[95% CI]	AME	[95% CI]
**Sex (ref. male)**										
Female	-0.2	[-0.5;0.1]	**-0.9**	[-1.1;-0.6]	0.1	[-0.1;0.3]	-0.2	[-0.3;0.0]	**1.1**	[0.9;1.3]
**Age group (ref. 18–24)**										
25–34	**-2.4**	[-2.9;-1.9]	0.0	[-0.3;0.3]	**1.9**	[1.7;2.1]	**0.6**	[0.3;0.8]	0.0	[-0.4;0.3]
35–44	**-3.3**	[-3.9;-2.7]	**1.1**	[0.6;1.5]	**2.0**	[1.6;2.3]	**0.8**	[0.5;1.1]	**-0.5**	[-0.9;-0.1]
45–54	**-2.3**	[-3.0;-1.7]	**2.5**	[2.0;3.0]	**1.4**	[1.0;1.7]	0.0	[-0.3;0.4]	**-1.6**	[-2.0;-1.2]
55–64	**-0.9**	[-1.6;-0.2]	**4.5**	[3.9;5.0]	**-0.3**	[-0.6;-0.0]	**-0.9**	[-1.2;-0.6]	**-2.3**	[-2.7;-1.9]
**Marital status (ref. married)**										
Never married or unknown	**-0.9**	[-1.3;-0.5]	**1.0**	[0.6;1.3]	**-0.8**	[-1.0;-0.5]	0.1	[-0.2;0.3]	**0.6**	[0.4;0.8]
Divorced or widowed	**-1.1**	[-1.7;-0.6]	**0.5**	[0.1;1.0]	**-0.4**	[-0.7;0.0]	**0.6**	[0.3;0.9]	**0.4**	[0.1;0.7]
**Level of education (ref. tertiary)**										
Secondary	**1.8**	[1.4;2.2]	**-0.7**	[-1.0;-0.4]	-0.1	[-0.4;0.1]	0.2	[0.0;0.5]	**-1.1**	[-1.4;-0.9]
Basic	-0.2	[-0.7;0.3]	**-1.3**	[-1.7;-0.9]	0.2	[-0.1;0.5]	**1.8**	[1.5;2.1]	**-0.5**	[-0.9;-0.2]
**Occupational class** **(ref. upper non-manual employees)**										
Lower non-manual employee	0.3	[-0.4;1.0]	0.0	[-0.7;0.7]	0.1	[-0.3;0.5]	0.3	[-0.4;1.0]	**-0.7**	[-1.1;-0.4]
Manual worker	**0.9**	[0.2;1.7]	0.6	[-0.1;1.3]	0.0	[-0.4;0.4]	-0.1	[-0.7;0.6]	**-1.5**	[-1.9;-1.1]
Entrepreneur	**2.3**	[1.0;3.6]	**-1.5**	[-2.8;-0.2]	-0.2	[-0.9;0.6]	-0.2	[-1.3;0.8]	-0.3	[-1.0;0.3]
Unemployed	**-7.5**	[-8.3;-6.7]	**8.2**	[7.5;8.9]	0.0	[-0.4;0.4]	0.1	[-0.5;0.8]	**-0.8**	[-1.4;-0.3]
Student	**-1.6**	[-2.4;-0.8]	**-3.1**	[-3.8;-2.4]	**5.2**	[4.8;5.7]	-0.4	[-1.0;0.3]	-0.2	[-0.7;0.3]
Other	**1.6**	[0.8;2.4]	**-3.7**	[-4.4;-3.0]	**0.6**	[0.2;1.1]	**1.9**	[1.2;2.5]	-0.4	[-0.9;0.1]
**Income** **(ref. highest quintile)**										
Quintile 4	-0.8	[-1.6;0.0]	-0.2	[-0.9;0.6]	**0.4**	[0.0;0.8]	0.5	[-0.3;1.2]	0.1	[-0.3;0.5]
Quintile 3	**-3.4**	[-4.2;-2.7]	**1.8**	[1.1;2.5]	**1.2**	[0.8;1.6]	0.4	[-0.2;1.1]	0.0	[-0.4;0.4]
Quintile 2	**-7.3**	[-8.1;-6.4]	**3.9**	[3.3;4.6]	**2.6**	[2.3;3.0]	**0.9**	[0.2;1.5]	-0.2	[-0.6;0.2]
Quintile 1 (lowest)	**-10.5**	[-11.4;-9.5]	**6.3**	[5.5;7.0]	**2.5**	[2.1;3.0]	**1.4**	[0.8;2.1]	0.2	[-0.3;0.7]
**Time in employment** **(ref. full year)**										
Less than a year	**-5.0**	[-5.5;-4.5]	**5.4**	[5.0;5.7]	**0.7**	[0.4;1.0]	**-0.9**	[-1.2;-0.6]	-0.2	[-0.5;0.1]
None	**-9.0**	[-9.7;-8.4]	**6.3**	[5.8;6.8]	**1.3**	[1.0;1.7]	**1.5**	[1.0;1.9]	0.0	[-0.4;0.4]
**Number of chronic diseases (ref: 0)**										
1	**-0.6**	[-1.1;-0.2]	0.1	[-0.3;0.5]	0.0	[-0.3;0.3]	**0.7**	[0.4;1.0]	-0.2	[-0.4;0.1]
2 or more	-0.3	[-0.8;0.3]	-0.1	[-0.6;0.3]	-0.1	[-0.5;0.3]	0.3	[0.0;0.6]	0.2	[0.0;0.5]
**Receipt of benefits during the year (ref. no receipt of the benefit)**										
Unemployment benefit	**-24.2**	[-24.9;-23.6]	**16.1**	[15.5;16.6]	**6.2**	[5.8;6.5]	**2.6**	[2.2;2.9]	**-0.5**	[-0.8;-0.2]
Disability or old-age pension	**-3.0**	[-3.9;-2.2]	**-1.5**	[-2.1;-0.9]	**-2.3**	[-2.6;-2.0]	**2.2**	[1.7;2.7]	**4.6**	[3.8;5.3]
Sickness allowance	**-1.8**	[-2.3;-1.2]	0.0	[-0.4;0.4]	**-0.9**	[-1.1;-0.6]	0.0	[-0.2;0.3]	**2.6**	[2.2;3.0]
Disability allowance	**-21.6**	[-23.3;-19.9]	**-1.1**	[-1.9;-0.2]	**-1.0**	[-1.6;-0.4]	**5.4**	[4.8;6.0]	**18.3**	[16.7;19.9]
Housing support	**-1.9**	[-2.3;-1.5]	**-0.6**	[-0.9;-0.3]	0.1	[-0.1;0.4]	**1.9**	[1.6;2.1]	**0.5**	[0.2;0.7]
Basic income support	**-6.3**	[-6.9;-5.8]	**2.0**	[1.6;2.4]	**0.7**	[0.4;1.0]	**3.9**	[3.6;4.3]	-0.3	[-0.6;0.0]

Adjusted average marginal effects (AME) and their 95% confidence intervals from fully adjusted multinomial logistic regression models. N = 119,740.

The likelihood to be assigned to cluster 2 increased especially by having older age (especially being over 55 years old), being unemployed by occupational class, having lower income, not being a full year in employment, and by being a recipient of unemployment benefit. The likelihood was decreased for those who were students or whose occupational class could not be defined (‘others’), at the beginning of the study years. In contrast, students and those having an occupational class of ‘others’ had an increased likelihood to be assigned to cluster 3. Assignment to cluster 3 was also increased especially for 25–44-year-olds and for those with lower income, those not employed for the whole year, and recipients of unemployment benefits.

The likelihood of being in cluster 4 was increased by being in age groups of 25–44 years and decreased by being in the age group 55–64, and also increased by being divorced or widowed, having only basic education, being in the occupational class ‘other’, having low income, not being in employment during the year, having chronic disease and being recipient of different benefits, especially disability allowance and basic income support.

Finally, the likelihood of being assigned to cluster 5 increased by being female, by not being currently married, and being recipient of benefits–especially disability allowance and disability or old-age pension. The likelihood to be assigned to this cluster decreased by older age, lower education and having an occupational status of a non-manual employee, manual worker or unemployed.

## Discussion

### Main findings

Understanding patterns of individuals’ use of healthcare services as well as social and employment services is important for designing functional, effective and coordinated service systems and care packages. However, we are not aware of previous studies combining individual-level healthcare utilization data with social and employment services utilization data in a comprehensive manner. This register-based study explored the use of 22 different healthcare, social and employment service types in a working-age population and strived to unravel different profiles in the population in terms of the individuals’ use of different types of services during one year. Utilizing K-means cluster analysis, five clusters with distinct profiles in terms of service use were identified. The clusters differed from each other in terms of unique patterns of service use and socio-demographic and morbidity-related predictors.

### Interpretation of the results

Our findings corroborate the results of previous studies finding that while the majority of the population are low-need service users, several clusters with use of more specialized services or intensive use profiles may be identified [1,16,17,19]. Our study found a large cluster (*low to moderate users of healthcare*, 82% of the working-age population) that had a lower than average level of public healthcare services use and very little social or employment services use. The adjusted likelihood of being assigned to this cluster was increased by, e.g., having younger age, more stable attachment to work, having higher income and not receiving social security benefits. This largest cluster included both those not using any of the studied services, as well as those with some use of healthcare. Healthcare services used in this cluster were mainly outpatient primary healthcare services.

This cluster often used occupational healthcare services and more rarely public sector services. The fact that persons assigned to this cluster most often used occupational healthcare is closely related to the fact that the majority of them were employed the full year, and OHC services were thus accessible for them. Also, it is likely that health problems of persons assigned to this cluster were most often not severe, and many of the OHC visits were probably related to mild issues such as need for sickness absence certification [34]. OHC may take care of easily treatable health issues under the primary healthcare contracts, whereas those in need of specialized healthcare are referred to specialized services at the public sector [25]. Also the use of private services was more frequent in this cluster than in the other clusters. Overall, persons in this large cluster seemed to have a favorable socio-economic position, lower-than-average healthcare needs and little social problems. The results are in line with previous research on the associations of socio-economic covariates with the intensity of healthcare services use [e.g., 12]. While this cluster consists of persons with a better-than-average position in the society and thus also fewer serious health and social problems, efforts related to prevention and service targeting should be put also on this group. Carefully designed preventive actions targeted for the grand mass of low-need persons may help prevent more serious problems from occurring later on and thus save costs in the long run [1].

While the largest cluster was similar to the large group of non-frequent healthcare users found also in other studies, our ability to also use data on social services and employment services was rather crucial in determining the remaining cluster groups. The second largest cluster, *regular employment services users with moderate use of healthcare* (10% of the study population) and the third largest cluster, *supported employment services users with moderate use of healthcare with an emphasis on preventive care* (3%), were most clearly distinguished by the fact that all persons assigned to these clusters had used either regular employment services (cluster 2) or supported employment services (cluster 3). Persons in clusters 2 and 3 used public healthcare clearly more often than those in cluster 1, and the third cluster was also distinctive in using a lot of preventive healthcare services. On the other hand, as those in clusters 2 and 3 used occupational healthcare and private healthcare less often, their overall frequency of using primary outpatient healthcare did not differ markedly from that of cluster 1.

Even though those assigned to clusters 2 and 3 were in many terms fairly similar based on their service use, the associations with covariates were somewhat different. Assignment to cluster 2 was predicted by, for example, older age (especially being in the age group 55–64), not being currently married and being unemployed by occupational class at the start of the year, while assignment to cluster 3 was predicted by being younger (especially being in the age group 25–44), being married and being a student by occupational class. Thus, it seems that younger persons may have been more intensively directed to the supported employment services, while less employment efforts have been put on older unemployed persons who are thus customers of regular employment services. The differences in socio-economic background also shed light on the differences in preventive healthcare use between clusters 2 and 3: the younger persons in cluster 3 may have used, for example, preventive student healthcare and maternity clinic services that are examples of services included in the category of preventive outpatient healthcare. To our knowledge, similar clusters as our clusters 2 and 3 have not been highlighted in previous studies on clustering of service users since, to our knowledge, data on employment services have not been linked to data on other services in a similar manner.

The fourth cluster identified in this study (*frequent users of healthcare*, *social and employment services*, 3% of the study population) consisted of persons with intensive use of public healthcare services, social services and employment services. This cluster stood out from the other clusters also through the fact that all persons in this cluster were customers of social work services, on average for almost the full year. While we did not focus on actual costs of care and did not define the study population directly by the frequency of visits but rather through latent grouping, this cluster resembles the group often called frequent users or high-cost users, that has been found in many previous studies examining healthcare use in Western countries [2,4–14]–albeit previous studies have tended to focus on healthcare services only. The probability to be assigned to this cluster was strongly associated with having a less advantaged socio-demographic position, which corroborates results of previous studies on high-cost or frequent healthcare attenders [2,12–15]. Our results support the interpretation that poor health may not be the only problem in this group, but the group is faced with intertwinement of poor health with other multifaceted issues, social exclusion and employability problems. While some studies have found that older age and female sex increase the likelihood of being a frequent or high-cost user [e.g., 2,7,13], our adjusted results showed no differences between men and women, and a smaller predicted probability was found for the oldest age group compared to younger age groups. However, the results are not directly comparable as previous studies have examine only healthcare services while our analyses included also social and employment services. This group of intensive service users, needing both healthcare services as well as social and employment services, is a natural target for interventions and service coordination and integration, which may both save considerable costs and help the individuals in coping with their multifaceted health issues and social problems. It has also been suggested that among frequent users, there are often unmet care needs and, on the other hand, the care received may be unnecessary or ineffective [10]. It seems evident that this group would greatly benefit from well-coordinated and integrated healthcare, social and employment services.

The fifth cluster identified in this study (*rehabilitation*, *disability services and specialized healthcare users*, 3% of the study population) consisted largely of persons using specialized healthcare services, rehabilitation services and services for the disabled–but not much employment services. This cluster seemed to consist of persons whose main issues seemed to be related to coping with their disability or other special health needs. Many were also occupational healthcare users, meaning that they have a contact to the working life. One third had visited private doctors, which indicates both high needs and the need to seek second opinions, as well as ability or willingness to pay high out-of-pocket payments. Adjusted for all covariates, assignment to this cluster was more likely among females, younger persons, those currently not married, those with tertiary education and those who were upper non-manual employees. This result partly corroborates previous Finnish research showing that adjusted for health status, those with higher education are more likely to participate in rehabilitation compared to those with low education [35]. The result may indicate that those with higher qualifications and higher social position may have been more able to navigate the care system and seek the services that they need to cope with their disability.

### Methodological considerations

A main strength of this study was the possibility to analyze a rich register data set that included information on various healthcare services as well as social and employment services in the working-age population of one municipality in Finland, including more than 100,000 study participants. Such a varied register data set has only rarely been available for research. Register data are generally of high quality, and there is no self-report bias and very little missing data. By combining comprehensive information on use of services with background variables, we were able to identify subgroups of service users and their predictors.

However, the study is not without limitations. We used data on the population of one municipality in Finland, a country with a comprehensive social insurance system and full coverage of public services. Thus, generalizability of the results to other countries with different service systems may be limited. Furthermore, even though we could measure long-term chronic diseases as a proxy for care needs, the limited number of health-related covariates in registers prevented us from taking need of services into account in a more comprehensive manner. Also, our data did not include information on costs, while this is an important aspect for financial and planning purposes: high attendance may not always mean high costs, if only low-cost services are used. Future studies should strive to assess joint costs of healthcare, social and employment services at the individual level. Furthermore, this study analyzed use of services among working-age persons only. Future studies should analyze the clustering of service use also among children and older people since their service needs and service packages are inherently different from those of the working-age population.

Another methodological issue is that the results of cluster analysis are dependent on the choices made by the researchers, as there are no clear and objective decision criteria in what comes to the exact clustering method or the number of clusters to be chosen [29]. We strived to tackle this problem by calculating cluster solutions for different numbers of clusters and decided on the final solution supported by cluster metrics. Also, cluster analysis is an exploratory method that identifies latent groups including average user profiles, not clear-cut groups of service users, and also the internal homogeneity of the clusters may vary. An important topic for further studies would be to analyze temporal aspects of clustering: how persistent are the clusters from year to year, how do people move between clusters in time, and what characteristics are associated with these changes? Finally, our results are descriptive associations with cross-sectional data, and thus we cannot infer any causal chains between the covariates and cluster membership or service use based on these analyses.

### Practical implications

Knowledge on the different user groups and their socio-demographic determinants helps in predicting the need of services, designing effective interventions and planning care management and coordinated services for persons with different needs. Better fitting services for the customer also offer potential for saving costs if persons in need are seamlessly guided to navigate between services relevant to them. Furthermore, case management across the service system could help reduce use and thus also cut costs [36,37]. Also knowledge on the predictors of the different user profiles may help in predicting future use of services as well as designing early interventions to prevent persons from moving from light use to more intense service use.

However, a commonly noted problem in the service system is that the services are organized separately at different schemes and by different providers, and coordination across the organizational borders is difficult. Our results call for the need of integration and coordination across traditionally separate actors in the service system, as health problems, social problems and unemployment are often intertwined. Our study was set in a time when public healthcare services and social services in Finland were organized by the municipalities (until end of year 2022). However, a major health and social services reform has taken place from the beginning of year 2023. Now, the responsibility for organizing healthcare and social services has been transferred from the municipalities to larger areas, i.e. to 22 self-governing wellbeing services counties that are responsible for organizing services within their own areas [26]. The purpose of the reform was to improve the availability and quality of basic public services, to achieve a more efficient care system, to offer better integrated healthcare and social services, and to contain costs [26]. However, even this reforms do not encompass all schemes and service providers–for example occupational healthcare, private healthcare and employment services are still organized separately from public healthcare and social services, and services cannot thus be directly coordinated between these actors.

In a scattered system, identification of user profiles and a functional coordination of all schemes, service types and providers would demand common patient registers and customer data systems, which are difficult to achieve under the current strict data protection regulations and with uncoordinated information systems. We succeeded to combine the different data sources only for research purposes. In order to achieve well-coordinated services, all relevant data scattered in different databases should be easily available to the professionals who are responsible for organizing and providing the services.

## Conclusions

This study identified five groups among working-age individuals in terms of their use of healthcare, social and employment services. While the majority of the population was classified into a cluster of low or moderate users of healthcare services, the study also found smaller clusters who had more specific patterns of service use and whose service combinations often also included social and employment services. In addition to being different in terms of their service use, these five clusters also had clearly different profiles in terms of socio-demographic covariates. Knowledge on the different user profiles and their determinants may help predict future need and use of services in different population groups, coordinate care, build effective and integrated multi-professional service combinations, and design early interventions and prevention measures in order to save costs and improve the overall quality and effectiveness of services.

## Supporting information

S1 Table Clustering metrics, K-means cluster analysis for solutions with 2–6 clusters. (DOCX)

S2 Table Distributions of the covariates by clusters. (DOCX)
