# Supplementary material for: Identifying user profiles of healthcare, social and employment services in a working-age population: A cluster analysis with linked individual-level register data from Finland
Source: PLoS One. 2023 Nov 1;18(11):e0293622. doi: 10.1371/journal.pone.0293622 (PMC10619802; doi:10.1371/journal.pone.0293622)
Supplement: S1 Table — (DOCX) [file pone.0293622.s001.docx]

**S1 Table.** **Clustering metrics, K-means cluster analysis for solutions with 2–6 clusters.**

| **Index** | **2 clusters** | **3 clusters** | **4 clusters** | **5 clusters** | **6 clusters** |
| --- | --- | --- | --- | --- | --- |
| Average silhouette width ^1^ | 0.64 | 0.66 | 0.68 | 0.70 | 0.63 |
| Calinski–Harabasz Index ^2^ | 50,876 | 49,302 | 45,436 | 46,837 | 43,468 |

^1^ Average silhouette width: a higher value indicates better clustering solution. ^2^ Calinski–Harabasz Index: a higher value indicates better clustering solution.
