# Supplementary material for: Identifying user profiles of healthcare, social and employment services in a working-age population: A cluster analysis with linked individual-level register data from Finland
Source: PLoS One. 2023 Nov 1;18(11):e0293622. doi: 10.1371/journal.pone.0293622 (PMC10619802; doi:10.1371/journal.pone.0293622)
Supplement: S2 Table — (DOCX) [file pone.0293622.s002.docx]

**S2 Table.** **Distributions of the covariates by clusters.^1^**

| **Variable ^2^** | **Cluster 1** | **Cluster 2** | **Cluster 3** | **Cluster 4** | **Cluster 5** |
| --- | --- | --- | --- | --- | --- |
|  | % | % | % | % | % |
| **Sex** |  |  |  |  |  |
| Male | 51.4 | 54.7 | 47.1 | 54.3 | 38.7 |
| Female | 48.6 | 45.3 | 52.9 | 45.7 | 61.3 |
| **Age group** |  |  |  |  |  |
| 18–24 | 16.3 | 17.7 | 18.9 | 24.1 | 20.0 |
| 25–34 | 22.7 | 25.6 | 40.7 | 27.9 | 24.9 |
| 35–44 | 22.4 | 18.6 | 23.3 | 19.3 | 21.8 |
| 45–54 | 19.6 | 18.4 | 13.2 | 14.8 | 15.5 |
| 55–64 | 19.0 | 19.6 | 3.8 | 13.7 | 17.8 |
| **Marital status** |  |  |  |  |  |
| Married | 43.8 | 27.1 | 32.6 | 16.1 | 26.2 |
| Never married or unknown | 45.0 | 59.2 | 58.5 | 67.9 | 61.3 |
| Divorced or widowed | 11.2 | 13.7 | 8.9 | 15.9 | 12.5 |
| **Level of education** |  |  |  |  |  |
| Tertiary | 43.9 | 25.5 | 23.4 | 9.9 | 35.8 |
| Secondary | 46.3 | 55.9 | 48.4 | 40.2 | 37.6 |
| Basic | 9.8 | 18.6 | 28.1 | 50.0 | 26.6 |
| **Occupational class** |  |  |  |  |  |
| Upper non-manual employee | 22.8 | 3.4 | 3.9 | 1.7 | 14.2 |
| Lower non-manual employee | 29.1 | 6.5 | 7.9 | 4.8 | 17.4 |
| Manual worker | 19.5 | 7.4 | 7.2 | 4.7 | 5.7 |
| Entrepreneur | 5.5 | 0.6 | 0.8 | 0.6 | 2.2 |
| Unemployed | 3.0 | 70.3 | 27.4 | 22.8 | 4.1 |
| Student | 9.8 | 7.4 | 45.4 | 17.2 | 14.7 |
| Other | 10.3 | 4.4 | 7.5 | 48.1 | 41.6 |
| **Income (during 2018)** |  |  |  |  |  |
| Quintile 5 (highest | 23.9 | 1.7 | 0.7 | 0.7 | 8.8 |
| Quintile 4 | 23.5 | 3.6 | 2.1 | 1.5 | 11.5 |
| Quintile 3 | 22.0 | 12.2 | 9.4 | 3.8 | 14.6 |
| Quintile 2 | 17.0 | 36.9 | 40.6 | 24.6 | 25.4 |
| Quintile 1 (lowest) | 13.7 | 45.7 | 47.3 | 69.3 | 39.7 |
| **Time in employment (during 2018)** |  |  |  |  |  |
| Full year | 66.9 | 5.5 | 8.1 | 7.1 | 32.3 |
| Less than a year | 20.4 | 45.2 | 46.3 | 12.9 | 17.6 |
| None | 12.8 | 49.3 | 45.6 | 80.0 | 50.1 |
| **Number of chronic diseases (during 2018)** |  |  |  |  |  |
| 0 | 79.2 | 78.4 | 84.8 | 61.8 | 57.2 |
| 1 | 13.1 | 13.0 | 10.2 | 20.8 | 19.6 |
| 2 or more | 7.7 | 8.6 | 5.0 | 17.4 | 23.3 |
| **Receipt of benefits (during 2018) ^3^** |  |  |  |  |  |
| Unemployment benefit (yes) | 9.0 | 93.7 | 85.8 | 47.5 | 9.6 |
| Disability pension or old-age pension (yes) | 10.1 | 5.2 | 0.8 | 36.3 | 49.1 |
| Sickness allowance (yes) | 9.2 | 9.8 | 6.5 | 12.1 | 17.3 |
| Disability allowance (yes) | 1.2 | 1.6 | 0.9 | 27.6 | 43.3 |
| Housing support (yes) | 18.7 | 53.3 | 59.9 | 84.3 | 46.6 |
| Basic income support (yes) | 3.2 | 30.8 | 33.0 | 56.3 | 9.5 |
| **Total** | 100 | 100 | 100 | 100 | 100 |
| **N** | 98,215 | 11,510 | 3,449 | 3,433 | 3,133 |

^1^ All associations are significant at the p<0.001 level. ^2^ Variables were measured at the beginning of year 2018 if not stated otherwise. ^3^ Proportions are given separately for receipt of each benefit.
